# Relative Impact of Multimorbid Chronic Conditions on Health-Related Quality of Life – Results from the MultiCare Cohort Study

**DOI:** 10.1371/journal.pone.0066742

**Published:** 2013-06-24

**Authors:** Christian Brettschneider, Hanna Leicht, Horst Bickel, Anne Dahlhaus, Angela Fuchs, Jochen Gensichen, Wolfgang Maier, Steffi Riedel-Heller, Ingmar Schäfer, Gerhard Schön, Siegfried Weyerer, Birgitt Wiese, Hendrik van den Bussche, Martin Scherer, Hans-Helmut König, the MultiCare Study Group, Attila Altiner, Horst Bickel, Wolfgang Blank, Christian Brettschneider, Monika Bullinger, Hendrik van den Bussche, Anne Dahlhaus, Lena Ehreke, Michael Freitag, Angela Fuchs, Jochen Gensichen, Ferdinand Gerlach, Heike Hansen, Sven Heinrich, Susanne Höfels, Olaf von dem Knesebeck, Hans-Helmut König, Norbert Krause, Hanna Leicht, Melanie Luppa, Wolfgang Maier, Manfred Mayer, Christine Mellert, Anna Nützel, Thomas Paschke, Juliana Petersen, Jana Prokein, Steffi Riedel-Heller, Heinz-Peter Romberg, Ingmar Schäfer, Martin Scherer, Gerhard Schön, Susanne Steinmann, Sven Schulz, Karl Wegscheider, Klaus Weckbecker, Jochen Werle, Siegfried Weyerer, Birgitt Wiese, Margrit Zieger

**Affiliations:** 1 Department of Health Economics and Health Services Research, Hamburg Center for Health Economics, University Medical Center Hamburg-Eppendorf, Hamburg, Germany; 2 Department of Psychiatry, Technical University of Munich, Munich, Germany; 3 Institute of General Practice, Goethe-University Frankfurt on the Main, Frankfurt on the Main, Germany; 4 Department of General Practice, University of Düsseldorf Medical Center, Düsseldorf, Germany; 5 Department of General Practice, Jena University Hospital, Jena, Germany; 6 Department of Psychiatry and Psychotherapy, University of Bonn, Bonn, Germany; 7 Institute of Social Medicine, Occupational Health and Public Health, University of Leipzig, Leipzig, Germany; 8 Department of Primary Medical Care, University Medical Center Hamburg-Eppendorf, Hamburg, Germany; 9 Department of Medical Biometry and Epidemiology, University Medical Center Hamburg-Eppendorf, Hamburg, Germany; 10 Central Institute of Mental Health, Medical Faculty Mannheim/Heidelberg University, Mannheim, Germany; 11 Institute for Biometry, Hannover Medical School, Hannover, Germany; INRCA, Italy

## Abstract

**Background:**

Multimorbidity has a negative impact on health-related quality of life (HRQL). Previous studies included only a limited number of conditions. In this study, we analyse the impact of a large number of conditions on HRQL in multimorbid patients without preselecting particular diseases. We also explore the effects of these conditions on the specific dimensions of HRQL.

**Materials and Methods:**

This analysis is based on a multicenter, prospective cohort study of 3189 multimorbid primary care patients aged 65 to 85. The impact of 45 conditions on HRQL was analysed. The severity of the conditions was rated. The EQ-5D, consisting of 5 dimensions and a visual-analogue-scale (EQ VAS), was employed. Data were analysed using multiple ordinary least squares and multiple logistic regressions. Multimorbidity measured by a weighted count score was significantly associated with lower overall HRQL (EQ VAS), b = −1.02 (SE: 0.06). Parkinson’s disease had the most pronounced negative effect on overall HRQL (EQ VAS), b = −12.29 (SE: 2.18), followed by rheumatism, depression, and obesity. With regard to the individual EQ-5D dimensions, depression (OR = 1.39 to 3.3) and obesity (OR = 1.44 to 1.95) affected all five dimensions of the EQ-5D negatively except for the dimension anxiety/depression. Obesity had a positive effect on this dimension, OR = 0.78 (SE: 0.07). The dimensions “self-care”, OR = 4.52 (SE: 1.37) and “usual activities”, OR = 3.59 (SE: 1.0), were most strongly affected by Parkinson’s disease. As a limitation our sample may only represent patients with at most moderate disease severity.

**Conclusions:**

The overall HRQL of multimorbid patients decreases with an increasing count and severity of conditions. Parkinson’s disease, depression and obesity have the strongest impact on HRQL. Further studies should address the impact of disease combinations which require very large sample sizes as well as advanced statistical methods.

## Introduction

In ageing societies multimorbidity is becoming an increasingly common phenomenon. Multimorbidity refers to the coexistence of more than one chronic condition in an individual, a phenomenon much more common in subjects aged 65 years and above than in younger subjects [1], [2]. Rates of prevalence vary across studies, owing to methodological differences. Since there is no uniform cut-off point for multimorbidity across studies, both two or more and three or more conditions are common criteria [3]. Also, rates of prevalence were found to be higher in primary-care based study populations than in samples drawn from the general population [4], and may depend on the number of conditions taken into consideration [5]. In a German study based on claims data from a large sample, van den Bussche et al. [6] found a prevalence of 62.1% for multimorbidity, defined as three or more conditions, among subjects aged 65 years or older and a mean number of 5.8 chronic conditions among these multimorbid subjects.

In the current study, our interest is in the impact of coexisting chronic conditions on health-related quality of life (HRQL). Multimorbidity, measured as the number of conditions, has been extensively shown to have a negative impact on HRQL [3], [7], which is more pronounced if disease severity is taken into account [8]. However, understanding the effects of multimorbidity in detail is not straightforward. First, individual chronic conditions may vary in their impact on patients’ daily lives and functioning [9], [10]. Moreover, different conditions may combine to produce subadditive, additive or superadditive effects on HRQL [11]. For instance, Hunger et al. [12] reported superadditive effects on HRQL of diabetes combined with a coronary event on the one and of a coronary event combined with stroke on the other hand, compared to the individual effects of these conditions. Also, conditions may affect separate dimensions of HRQL differently. Hodek et al. [13], for example, found that peripheral arterial disease (PAD) and both cardiovascular and cerebrovascular events had a negative effect on overall HRQL as measured by the EQ-5D. While cardiovascular events affected HRQL across all EQ-5D dimensions, PAD had effects on mobility and pain/discomfort, and cerebrovascular events had effects on self-care and anxiety/depression only.

Finally, the number of potential combinations of diseases is virtually limitless [6]. To separate out the effects of individual conditions on HRQL, a large number of potentially relevant conditions has to be controlled for. Previous studies that analysed the impact of specific multimorbid conditions or their combination on HRQL are based on data which include only a limited number of conditions or their combinations, but no additional potentially comorbid diseases [12], [14], [15].

In this study, we take a somewhat different, bottom-up approach and analyse the relative impact of a large number of potentially coexisting conditions on overall HRQL in multimorbid patients without preselecting particular diseases. We also explore the effects of these different conditions on the specific dimensions of HRQL.

## Materials and Methods

### Ethics Statement

Written informed consent was obtained previous to inclusion into the study. The study was approved by the Ethics Committee of the Medical Association of Hamburg (Approval-No. 2881) and conducted according to the principles expressed in the declaration of Helsinki.

### Sample

Data were collected as part of the MultiCare Cohort Study. Details regarding the methods of the study and the cohort have been published elsewhere [16], [17]. The analyses presented here are based on data from the MultiCare baseline assessment. Briefly, the MultiCare Cohort Study is a multicentre, prospective cohort study of multimorbid primary care patients selected randomly from the databases of 158 general practitioner’s (GP) offices at 8 study centres across Germany. The study’s aims are the investigation of multimorbidity patterns over time, the identification of patients’ resources and risk factors that influence the course of these patterns, and the analysis of somatic, psychological and social consequences of these patterns for the patients’ quality of life and functional status. Inclusion criteria were multimorbidity, defined as the coexistence of at least 3 chronic conditions from a list of 29 diseases, age between 65 and 85 years, and at least one visit to the GP within the last completed quarter (three-month period). Patients were excluded from the study if they were no regular patients of the GP. Other exclusion criteria were inability to participate due to medical reasons (such as blindness and deafness), insufficient German language skills, residence in a nursing home, inability to provide written informed consent and participation in another ongoing study. A diagnosis of dementia was therefore an exclusion criterion.

A total of 24,862 patients from the databases of the participating GP practices were checked for inclusion and exclusion criteria. 7,172 patients fulfilled the criteria and were contacted for informed consent to participate. 3,317 patients agreed to participate and were available for the baseline interview within a time frame of 16 months. In retrospect, 128 of these cases had to be excluded either because in direct contact exclusion criteria were found to apply or because the patient died before the baseline interview. Thus, a final number of 3,189 patients were included in the study.

Recruitment and baseline interviews took place between July 2008 and October 2009. The study was approved by the Ethics Committee of the Medical Association of Hamburg.

### Sociodemographic Variables and Social Support

Socio-economic status (education, income) was assessed with an established questionnaire [18]. The level of education was rated according to the international CASMIN classification [19]. Income is reported as net income adjusted for household size (this is net income divided by the equivalised household size, for which a value of 1.0 is assigned to the householder, 0.5 is assigned to every other household member aged 15 or over and 0.3 is assigned to every child under the age of 15). Social support was assessed by the F-SOZU K-14 questionnaire [20], which comprises 14 items for emotional and practical support and social integration and yields an overall score between 1 and 5. A higher score means better social support.

### Multimorbidity

Multimorbidity was assessed by means of a standardised GP questionnaire which comprised 46 diagnosis groups. The list was newly compiled at the beginning of the MultiCare study with the aim of representing the most frequent chronic conditions in the population and is based on prevalence data [6], [16]. In the 46 groups, ICD-10 codes are classified together if diseases and syndromes are similar pathophysiologically or if ICD codes of related disorders are used ambiguously in practice. At the beginning of the baseline interviews, the compilation of the list had not been quite finalised, and for this reason seven of the diagnosis groups were not part of the standardised baseline GP questionnaire, but were assessed using responses to open questions. This applies to chronic gastritis/gastroesophageal reflux disease (GERD), insomnia, allergies, obesity, hypotension, sexual dysfunction and tobacco abuse, which may be subject to underreporting as a result.

The diagnosis groups assessed in a standardised fashion in the GP questionnaire at baseline include hypertension, lipid metabolism disorders, chronic low back pain, joint arthrosis, diabetes mellitus, thyroid dysfunction, chronic ischemic heart disease, cardiac arrhythmias, asthma/chronic obstructive pulmonary disease (COPD), lower limb varicosis, osteoporosis, severe vision reduction, cancers, depression, purine/pyrimidine metabolism disorders and gout, atherosclerosis/peripheral arterial occlusive disease (PAD), intestinal diverticulosis, neuropathies, cardiac insufficiency, cerebral ischemia/chronic stroke, prostatic hyperplasia, renal insufficiency, cardiac valve disorders, chronic cholecystitis/gallstones, dizziness, liver diseases, haemorrhoids, urinary incontinence, somatoform disorders, severe hearing loss, anemias, rheumatoid arthritis/chronic polyarthritis (rheumatism), anxiety disorders, psoriasis, migraine/chronic headache, noninflammatory gynaecological problems, Parkinson’s disease, and urinary tract calculi. Dementia was also listed, but constituted an exclusion criterion at baseline, reducing the list of diseases to 45. Conditions were rated as present or not and, if present, were given a severity rating accounting for the prognosis of the course of disease and for the burden of disease between 0 (insignificant) and 4 (very severe). From these data, a simple and a weighted count score were derived, with the weighted count score corresponding to the sum of the severity ratings.

### Quality of Life

Health-related quality of life (HRQL) was assessed by means of the EQ-5D descriptive system and the EQ-5D visual analogue scale (EQ-VAS) [21]. The descriptive system consists of five items for the dimensions mobility, self-care, usual activities, pain/discomfort and anxiety/depression, with response categories 1 (no problems), 2 (some problems) or 3 (severe problems). We used the EQ-VAS for an overall HRQL-score, ranging from 0 (worst imaginable health state) to 100 (best imaginable health state). We also evaluated the individual scores for the five separate items of the EQ-5D descriptive system. For use as dependent variables in multiple logistic regression these scores were dichotomised to obtain the values 0 (no problems) and 1 (moderate or severe problems).

### Missing Values

Missing values were imputed using the hot deck method, in which missing values are replaced using observed values from a responding unit that is as similar as possible to the non-responding unit [17], [22].

### Statistical Analyses

For the analyses of effects of multimorbidity and of individual conditions on overall HRQL and on specific dimensions, multiple ordinary least squares (OLS) regression and multiple logistic regression were used. All models controlled for the same set of sociodemographic variables (age, gender, marital status, living situation, education, net income adjusted for household size) and for social support.

In a first model, multiple ordinary least squares (OLS) regression was used to estimate the effect of multimorbidity, as measured by the weighted count score, on overall HRQL as expressed by the EQ-VAS. Another multiple OLS model analysed the effects of individual conditions (coded as present or absent) on HRQL. Conditions with a prevalence of <1% in the sample (corresponding to less than 30 individuals concerned) were excluded in order to avoid problems resulting from insufficient cell size. This applied to hypotension, sexual dysfunction and tobacco abuse. For the regression analyses, the obesity item from the GP questionnaire, which was likely to be affected by underreporting (see above), was replaced by a dichotomous variable for obesity derived from the patients’ body mass index (BMI) using the WHO definition of obesity (BMI ≥30) [23].

A first model including all 42 conditions with a prevalence of ≥1% resulted in a large number of conditions without significant effect on HRQL. Therefore a reduced model included only those predictors with a *p* value of <0.05, and a nested model F-test was used to check whether the full model had greater explanatory power than the reduced model. Since this was not the case, the reduced model was selected over the full model.

In the second part of the analyses, multiple logistic regression models were estimated to explore the effects of individual conditions on the five specific dimensions of HRQL using dichotomised responses to the respective EQ-5D dimension as the dependent variable. Again, the five initial models contained a large number of insignificant variables, thus the same selection strategy as described for the OLS model above was applied to limit the list of independent variables to those with predictive value for the dependent variable, resulting in five reduced models with different sets of predictors.

A p-value <0.01 was considered as statistically significant. Statistical analysis was performed using STATA Release 11 (Stata Corp., College Station, Texas).

## Results

### Sociodemographic Data

The mean age of the sample at baseline was 74.4 years, and 59.3% were female. Over half of the participants were married (56.2%), while approximately a quarter were widowed (27.7%). 57.9% were living with their spouse or partner and 35.4% were living alone in their own home. The proportions of subjects in assisted living (1.7%) or retirement homes (0.3%) were low. The majority of the sample had a low degree of education (62.3%), and mean household-size adjusted income was 1,412 € (see Table 1).

**Table 1:** Sociodemographics characteristics and morbidity.

Characteristic	n (3,189)	%
Mean age (SD)	74.4 (5.2)	
Sex		
Female	1,891	59.3
Male	1,298	40,7
Marital status^a^		
Single	188	5.9
Married	1,791	56.2
Married, but separated	72	2.3
Divorced	255	8.0
Widowed	882	27.7
Living situation		
Alone	1,128	35.4
With spouse/partner	1,847	57.9
With family members	132	4.1
With others	21	0.7
Assisted living	53	1.7
Retirement home	8	0.3
Education		
Low	1,986	62.3
Medium	856	26.8
High	347	10.9
Mean income (SD)^b^ [€]	1,412 (704)	
Multimorbidity: mean (SD)		
Number of conditions	7.0 (2.5)	
Multimorbidity: mean (SD)		
Weighted count	11.2 (5.1)	
EQ-VAS: mean (SD)^c^	62.4 (18.2)	

a n = 3,188;

b n = 2,793;

c n = 3,178.

### Multimorbidity

On average, the participants had 7.0 chronic conditions (see Table 1), with no significant differences between men and women (data not shown). The ten most prevalent conditions in the overall sample, in descending order, were hypertension (77.9%), lipid metabolism disorders (58.5%), chronic low back pain (49.5%), joint arthrosis (43.3%), diabetes mellitus (37.6%), thyroid dysfunction (33.8%), chronic ischemic heart disease (31.4%), cardiac arrythmias (26.9%), asthma/COPD (24.2%) and lower limb varicosis (23.3%; see Table 2). However, there were some differences in rank order by gender. For instance, the prevalence of chronic ischemic heart disease is twice as high for men (44.7%) as for women (22.2%), for whom this condition only ranks twelfth. Osteoporosis, by contrast, was much more common among women, for whom this conditions ranks seventh, than men (28.9% vs. 6.6%). Among men thyroid dysfunction and lower limb varicosis are less common than among women (19.6% vs. 43.5% and 15.2% vs. 28.8%), while the ten most prevalent conditions for men also include prostatic hyperplasia (27.9%, ranking eighth) and purine/pyrimidine metabolism disorders and gout (23.7%, ranking tenth).

**Table 2:** Prevalence of diagnosis groups and rank order in the sample, overall and by gender.

Diagnosis group	Overall (n = 3,189)	Female (n = 1,891)	Male (n = 1,298)
Hypertension	77.9% (1)	77.5% (1)	78.4% (1)
Lipid metabolism disorders	58.5% (2)	57.0% (2)	60.8% (2)
Chronic low back pain	49.5% (3)	55.2% (3)	41.1% (5)
Joint arthrosis	43.3% (4)	48.9% (4)	35.3% (6)
Diabetes mellitus	37.6% (5)	33.3% (6)	43.8% (4)
Thyroid dysfunction	33.8% (6)	43.5% (5)	19.6% (13)
Chronic ischemic heart disease	31.4% (7)	22.2% (12)	44.7% (3)
Cardiac arrhythmias	26.9% (8)	23.5% (9)	31.9% (7)
Asthma/COPD	24.2% (9)	23.1% (10)	25.7% (9)
Lower limb varicosis	23.3% (10)	28.8% (8)	15.2% (17)
Osteoporosis	19.8% (11)	28.9% (7)	6.6% (27)
Severe vision reduction	18.9% (12)	19.5% (13)	18.2% (14)
Cancers	18.3% (13)	15.5% (14)	22.4% (12)
Depression	17.8% (14)	22.6% (11)	10.6% (22)
Purine/pyrimidine metabolism disorders/Gout	17.3% (15)	12.9% (18)	23.7% (10)
Atherosclerosis/PAOD	16.7% (16)	12.0% (19)	23.4% (11)
Intestinal diverticulosis	14.5% (17)	15.5% (14)	13.0% (20)
Neuropathies	14.7% (18)	13.0% (17)	17.3% (15)
Cardiac insufficiency	13.1% (19)	11.8% (20)	15.0% (19)
Chronic gastritis/GERD*	12.9% (20)	13.6% (16)	11.9% (21)
Cerebral ischemia/Chronic stroke	11.8% (21)	9.5% (22)	15.1% (18)
Prostatic hyperplasia	11.4% (22)	–	27.9% (8)
Renal insufficiency	10.7% (23)	7.1% (27)	15.8% (16)
Cardiac valve disorders	9.4% (24)	8.8% (23)	10.3% (23)
Chronic cholecystitis/Gallstones	7.9% (25)	8.3% (25)	7.2% (26)
Dizziness	7.7% (26)	8.7% (24)	6.3% (29)
Liver diseases	7.7% (27)	6.8% (28)	9.0% (25)
Haemorrhoids	7.5% (28)	5.6% (30)	10.3% (23)
Urinary incontinence	7.2% (29)	9.9% (21)	3.3% (36)
Somatoform disorders	6.1% (30)	7.7% (26)	3.7% (34)
Insomnia*	5.6% (31)	5.1% (33)	6.2% (30)
Severe hearing loss	5.2% (32)	4.2% (36)	6.8% (28)
Allergies*	4.9% (33)	6.0% (29)	3.4% (35)
Obesity*	4.8% (34)	4.8% (35)	4.9% (33)
Anemias	4.3% (35)	3.1% (38)	5.9% (31)
Rheumatoid arthritis/Chronic polyarthritis	4.2% (36)	5.6% (31)	2.2% (39)
Anxiety	4.1% (37)	5.3% (32)	2.2% (39)
Psoriasis	3.6% (38)	2.7% (39)	5.0% (32)
Migraine/chronic headache	3.5% (39)	4.9% (34)	1.5% (41)
Noninflammatory gynaecological problems	2.0% (40)	3.4% (37)	–
Parkinson’s disease	1.9% (41)	1.4% (40)	2.8% (37)
Urinary tract calculi	1.8% (42)	1.3% (41)	2.6% (38)
Hypotension*	0.5% (43)	0.5% (42)	0.4% (43)
Sexual dysfunction*	0.2% (44)	–	0.5% (42)
Tobacco abuse*	0.1% (45)	0 (43)	0.2% (44)
Dementias	–	–	–

* Not part of the GP questionnaire. Assessed using responses to open questions.

### EQ-5D

The average EQ-VAS score in the sample was 62.4 (see Table 1). There was a small but statistically significant difference, with higher EQ-VAS scores for men (mean 63.6, SD 18.4) than women (mean 61.6, SD 18.0; *p*<.01, two-tailed t test). Results for the EQ-5D descriptive system are displayed in Table 3. Half of the sample (49.4%) reported no problems with mobility, 90.5% reported no problems with regard to self care, and 71.6% reported no problems with usual activities and no anxiety or depression, respectively. Only 31.7% of the sample reported no pain/discomfort. There were significant gender differences in the proportions of the sample reporting no problems for all dimensions except self-care, with fewer women reporting no problems than men throughout (mobility: 47.3% of women vs. 52.4% of men, usual activities: 76.4% vs. 86.3%, pain: 26.4% vs. 39.4%, anxiety/depression: 64.8% vs. 81.5%, details not shown).

**Table 3:** Distribution of responses in the EQ-5D dimensions in the study sample.

EQ-5D item	n (%)		
	No problems	Some problems	Severe problems
EQ1: Mobility a	1,575 (49.4)	1,598 (50.1)	12 (0.4)
EQ2: Self-care b	2,886 (90.5)	259 (8.1)	43 (1.4)
EQ3: Usual activities	2,284 (71.6)	813 (25.5)	92 (2.9)
EQ4: Pain/discomfort b	1,010 (31.7)	1,689 (53.0)	489 (15.3)
EQ5: Anxiety/depression c	2,284 (71.6)	783 (24.6)	120 (3.8)

a n = 3,185;

b n = 3,188;

c n = 3,187.

### Effects of Multimorbidity and of Individual Conditions on Overall HRQL

The analysis of the effect of multimorbidity, as measured by the weighted count score, on HRQL indicates a highly significant relationship between greater morbidity and lower HRQL (see Table 4). The more detailed analysis of the impact of individual disease groups, coded as present vs. absent, on HRQL is presented in Table 5.

**Table 4:** Regression model of the effect of multimorbidity (weighted count score) on overall HRQL measured by the EQ VAS.

Variable	b (SE)	*p* value
**Weighted count ** ***(centered)***	**−1.02 (0.06)**	**0.000**
**Sex ** ***(Ref: male)***	**−1.97 (0.67)**	**0.003**
Age *(centered)*	−0.15 (0.61)	0.015
Marital Status *(ref: Married)*		
Married but separated	−2.53 (2.12)	0.233
Single	2.47 (1.74)	0.157
Divorced	−2.72 (1.56)	0.081
Widowed	−1.08 (1.33)	0.418
Living situation *(ref: Alone)*		
With spouse/partner	−1.41 (1.31)	0.280
With family members	0.85 (1.59)	0.591
With others	6.15 (3.76)	0.102
Assisted living	−3.80 (2.40)	0.114
Retirement home	−4.69 (6.03)	0.436
Level of education *(ref: low)*		
Medium	1.14 (0.70)	0.105
High	2.47 (1.04)	0.017
**Income (in 1000 €) ** ***(centered)***	**2.00 (0.4)**	**0.000**
**Social support ** ***(centered)***	**3.95 (0.45)**	**0.000**
**Intercept**	**66.21 (1.74)**	**0.000**

n = 3,189; Adj. R^2^ = 0.14; Significant results are printed bold.

**Table 5:** Multiple linear regression model of the effect of individual conditions on overall HRQL measured by EQ-VAS.

EQ-VAS	b (SE)	*p* value
**Age ** ***(centered)***	**−0.22 (0.06)**	**0.000**
Sex *(Ref.: male)*	−0.16 (0.73)	0.831
Marital Status *(ref: Married)*		
Separated	−1.37 (2.03)	0.500
** Single**	**4.40 (1.33)**	**0.001**
Divorced	−0.98 (1.16)	0.398
Widowed	0.50 (0.77)	0.516
Level of education *(ref: low)*		
Education: medium	1.09 (0.70)	0.122
** Education: high**	**3.23 (1.04)**	**0.002**
**Adj. income (1,000 €)**	**1.681 (0.45)**	**0.000**
**Social support**	**3.76 (0.45)**	**0.000**
		
**Parkinson’s disease**	**−12.29 (2.18)**	**0.000**
**Rheumatism/CPA**	**−5.56 (1.50)**	**0.000**
**Depression**	**−5.33 (0.80)**	**0.000**
**Obesity**	**−4.33 (0.66)**	**0.000**
**Cardiac insufficiency**	**−4.19 (0.92)**	**0.000**
**Neuropathies**	**−3.70 (0.85)**	**0.000**
**Asthma/COPD**	**−2.96 (0.71)**	**0.000**
**Osteoporosis**	**−2.92 (0.79)**	**0.000**
**Chronic low back pain**	**−2.70 (0.62)**	**0.000**
**Coronary heart disease**	**−2.47 (0.68)**	**0.000**
**Insomnia**	**−3.79 (1.32)**	**0.004**
**Urinary incontinence**	**−3.18 (1.17)**	**0.007**
Cardiac arrhythmias	−1.66 (0.69)	0.016
Joint arthrosis	−1.60 (0.62)	0.010
Lip. met. disord.	1.15 (0.62)	0.061
Allergies	3.22 (1.39)	0.021
**Intercept**	**69.74 (1.33)**	**0.000**

n = 3189; Adj. R^2^: 0.14; Significant results are printed bold.

Parkinson’s disease had the most pronounced negative effect on HRQL, as measured by the EQ-VAS (−12.29), followed by rheumatism/CPA (−5.56), depression (−5.33), obesity (−4.33) and cardiac insufficiency (−4.19). Neuropathies, asthma/COPD, osteoporosis, chronic low back pain, coronary heart disease, insomnia and urinary incontinence had significant negative effects on HRQL. In addition, higher age was associated with lower HRQL, while being single (as opposed to being married), a high education, income and social support were positively related to HRQL.

### Effects of Individual Conditions on Specific EQ-5D Dimensions

Table 6 summarizes the results of five logistic regression models for the effects of individual diseases on the occurrence of problems in the separate EQ-5D dimensions. The probability of moderate or severe problems with mobility is most markedly increased by the presence of obesity (OR 1.95), anemia (OR 1.79), joint arthrosis (OR 1.68), cardiac insufficiency (OR 1.63), chronic stroke (OR 1.53) and depression (OR 1.44). Furthermore atherosclerosis, neuropathy, asthma/COPD, diabetes mellitus and chronic low back pain were associated with problems in mobility. As regards the probability of problems with self-care, Parkinson’s disease has the greatest impact (OR 4.52), followed by chronic stroke (OR 2.04), urinary incontinence (2.01) cardiac insufficiency (OR 1.95), osteoporosis (OR 1.75), depression (OR 1.75), renal insufficiency (OR 1.70) and obesity (OR 1.69). Additionally neuropathy and chronic low back pain were related to problems in self-care.

**Table 6:** Logistic regression models of the effect of individual conditions on separate EQ-5D dimensions.

	Odds ratios *(SE)*				
	EQ-1: mobility	EQ-2: self-care	EQ-3: usual activities	EQ-4: pain/discomfort	EQ-5:anxiety/depression
Age	**1.05 (0.01)** **	**1.04 (0.01)** *	**1.04 (0.01)** **	1.01 (0.01)	0.99 (0.01)
Sex	1.13 (0.10)	0.90 (0.14)	**1.38 (0.14)** *	**1.51 (0.13)** **	**2.09 (0.19)** **
Living situation *(Ref.: Alone)*					
With spouse/partner		1.00 (0.15)	1.16 (0.11)		
With relatives		1.46 (0.41)	1.28 (0.26)		
With others		0.98 (0.68)	2.38 (1.14)		
Assisted living		**3.89 (1.31)** **	**2.24 (0.69)** *		
Retirement home		0.87 (1.03)	2.41 (1.84)		
Education *(Ref.: Low)*					
Medium			**0.76 (0.08)** *		
High			0.96 (0.14)		
Social support	**0.82 (0.05)** **	**0.63 (0.05)** **	**0.76 (0.05)** **	0.87 (0.05)	**0.62 (0.04)** **
Depression	**1.44 (0.15)** **	**1.75 (0.27)** **	**1.76 (0.19)** **	**1.39 (0.16)** *	**3.30 (0.33)** **
Obesity	**1.95 (0.17)** **	**1.69 (0.23)** **	**1.63 (0.15)** **	**1.44 (0.13)** **	**0.78 (0.07)** *
Chronic low back pain	**1.23 (0.10)** *	**1.54 (0.21)** *	**1.38 (0.12)** **	**1.66 (0.14)** **	
Neuropathies	**1.36 (0.15)** *	**1.59 (0.26)** *	**1.39 (0.16)** *	**1.40 (0.17)** *	
Anemias	**1.79 (0.34)** *		**1.89 (0.37)** *	**1.80 (0.38)** *	
Asthma/COPD	**1.31 (0.12)** *		**1.38 (0.13)** *	**1.49 (0.14)** **	
Cardiac insufficiency	**1.63 (0.19)** **	**1.95 (0.32)** **	**1.94 (0.23)** **		
Osteoporosis	1.28 (0.13)	**1.75 (0.28)** **	**1.39 (0.14)** *	**1.35 (0.15)** *	
Chronic stroke	**1.53 (0.18)** **	**2.04 (0.34)** **	1.38 (0.17)		
Joint arthrosis	**1.68 (0.13)** **		1.22 (0.10)	**1.64 (0.14)** **	
Lipid metabolism disorders	**0.76 (0.06)** **	**0.62 (0.08)** **	0.85 (0.07)		
Parkinson’s disease		**4.52 (1.37)** **	**3.59 (1.00)** **		2.11 (0.61)
Urinary incontinence		**2.01 (0.40)** *	**1.53 (0.23)** *		1.39 (0.21)
Anxiety disorders		0.48 (0.19)			**2.31 (0.46)** **
Atherosclerosis	**1.36 (0.14)** *				0.77 (0.09)
Cardiac valve disorders				**0.77 (0.10)** *	
Diabetes mellitus	**1.27 (0.10)** *	1.41 (0.19)			
Insomnia					**1.60 (0.28)** *
Renal insufficiency		**1.70 (0.31)** *	1.36 (0.18)		
Severe hearing loss				**0.63 (0.11)** *	
Coronary heart disease	1.24 (0.11)				
Dizziness					1.36 (0.20)
Intestinal diverticulosis					1.28 (0.15)
Psoriasis	1.53 (0.31)				
Rheumatism/CPA	1.57 (0.31)				
Urinary tract calculi			1.81 (0.53)		
Varicosis				0.80 (0.08)	

* p<0.01;

** p<0.001; Significant results are printed bold. All variables included in the second model are presented.

With respect to usual activities the probability of difficulties is most markedly increased by Parkinson’s disease (OR 3.59), cardiac insufficiency (OR 1.94), anemia (OR 1.89), depression (OR 1.76), obesity (OR 1.63) and urinary incontinence (OR 1.53). There were also associations between problems in usual activities and osteoporosis, neuropathy, chronic low back pain and asthma/COPD.

The association with moderate or severe pain or discomfort is greatest for anemia (OR 1.80), chronic low back pain (OR 1.66), joint arthrosis (OR 1.64), asthma/COPD (OR 1.49) and obesity (OR 1.44). Neuropathy, depression and osteoporosis were also related to problems in pain/discomfort.

Finally, there are only three conditions with significant effects on the dimension depression/anxiety. Depression had the greatest effect (OR 3.30), followed by anxiety disorders (OR 2.31) and insomnia (OR 1.60).

Across the five EQ-5D dimensions, there were individual positive effects on the outcome for lipid metabolism disorders (mobility, self-care), severe hearing loss (pain/discomfort) cardiac valve disorders (pain/discomfort) and obesity (anxiety/depression).

Apart from the effects of chronic conditions, there was an effect of higher age on the probability of difficulties in the dimensions mobility, self-care and usual activities. Female gender was associated with greater likelihood of problems with usual activities as well as pain or discomfort and depression or anxiety. Social support was a highly significant protective factor for all dimensions except pain/discomfort. Furthermore assisted living as compared to living alone was associated with problems in the dimension self-care (OR 3.89) and usual activities (OR 2.24). Compared to a lower level of education, a medium level was associated with fewer problems in the dimension usual activities. There were no other consistent effects of sociodemographic variables.

## Discussion

The aim of this study was to analyze the relative impact of multiple chronic conditions on HRQL in elderly patients. The patient cohort was recruited in general practices in eight German cities. Patients residing in nursing homes were excluded as well as those suffering from severe diseases likely to be fatal within three months according to the GP. Therefore our study sample may have missed some patients with higher disease severities. On average patients suffered from seven conditions and had a mean weighted multimorbidity count score of 11.2. This means that the average disease severity was 1.6. This indicates that patients were suffering from a greater number of less severe diseases but does not preclude that there were very severe disease affecting the patients life profoundly.

An advantage of our study compared to other studies of HROL in multimorbid patients is the great number of included conditions. We included 42 conditions whereas other studies considered only 6 [12] up to 29 conditions [24]. A greater number of conditions allows for a more comprehensive description of the influence of multimorbidity on HRQL. On the other hand the comparability of our results to the results of other studies is limited by the greater number of conditions.

In general, we found that multimorbidity measured by a weighted count score was negatively associated with overall HRQL measured by the EQ VAS. This is in line with findings of various other studies which also used the EQ-5D [14], [15], [24], [25], [26]. However, the patient samples of these studies differed with regard to age. While two study samples [14], [25] had a mean age (70 years) similar to our study, the studies of Agborsangaya et al. [26] and Saarni et al. [24] analysed samples with a mean age of approximately 50 years which indicates that the negative association of multimorbidity and overall HRQL can also be found in younger populations. Yet, all mentioned studies only used a simple count score to measure multimorbidity, whereas we additionally employed a severity weighting.

Focussing on the individual conditions we found 21 chronic conditions affecting the overall HRQL or the single dimensions of the EQ-5D. In general, Parkinsońs disease had the strongest effect on overall HRQL of the individual chronic conditions, followed by rheumatism, depression, cardiac insufficiency and obesity. With regard to the five dimensions of the EQ-5D, depression was significantly associated with problems across dimensions. Obesity, chronic low back pain and neuropathies were associated with greater problems in all dimensions but anxiety/depression. At the same time, obesity had a protective effect with regard to anxiety/depression. Cardiac insufficiency, anemia, asthma/COPD and osteoporosis were associated with three dimensions. Parkinsońs disease had the strongest negative impact (i.e. the largest OR) of all conditions on the EQ-5D dimensions self-care and usual activities. In the following section we will point out the most important of these chronic conditions as they influence overall HRQL and the dimensions of the EQ-5D in a substantial manner.

The effect of Parkinsońs disease is remarkable. It had the strongest influence of all chronic conditions on overall HRQL and the dimensions self-care and usual activities. The presence of Parkinsońs disease was associated with a mean loss of overall HRQL of more than 12 points on the EQ-VAS. Furthermore it increased the odds of reporting problems in self-care and usual activities by factors 4.5 and 3.6, respectively. In the literature reporting the effect of multimorbidity on HRQL, Parkinsońs disease has rarely been considered. One exception is the study by Saarni et al. who used the EQ-5D index in their analysis [24]. These authors also found that Parkinsońs disease had the strongest impact on HRQL of all 29 conditions included in their analysis.

Regarding the influence of Parkinsońs disease on the dimensions self-care and usual activities we found no comparable data from multimorbid populations in the literature. Winter et al [27] and Schrag et al [28] who assessed the impact of Parkinsońs disease on the EQ-5D dimensions without considering co-morbidity also found a higher proportion of patients reporting problems in these dimensions. Unfortunately it is not possible to compare their odds ratios with our findings as both studies compared their samples to younger general population samples.

The great impact of Parkinsońs disease on HRQL in multimorbid patients becomes obvious when compared to the next most influential conditions: chronic stroke (on self-care) and cardiac insufficiency (on usual activities). They show much smaller OR of 2.04 (compared to 4.52 for Parkinson’s disease) and 1.94 (compared to 3.59).

We found that obesity is another highly important factor for HRQL. The presence of obesity caused a decrease in overall HRQL of more than 4 points. This finding is in line with results reported by Hunger et al. from a population of multimorbid patients showing that HRQL decreases with increasing BMI after exceeding the threshold of 25 kg/m^2^ [12]. This is also reported by S?ltoft et al from a general population sample [29]. Furthermore the presence of obesity had an effect on the dimensions mobility, self-care and usual activities. Especially the effect on mobility was substantial with an OR of nearly 2. Overall, the OR found in our study were smaller in comparison to other studies. Agborsangaya et al. reported OR of 3.7 for mobility, 3.5 for usual activities and 2.9 for self-care [26]. Interestingly, the OR they reported are larger in general. Possible reasons for this are the mean age of their study sample and the number of chronic conditions considered. We analysed the impact of 42 conditions in sample with a mean age of 74.4 years whereas Agborsangaya et al. considered 16 conditions in a sample with a mean age of 46.6 years. Based on the observations that the number of diseases [24] and the number of reported problems [26] increases with increasing age, it is plausible that we found smaller OR in our study.

As expected, anxiety and depression had the strongest impact on the dimension Anxiety/Depression. The presence of these conditions was associated with comparatively large OR in our sample. Furthermore we found a strong impact of depression on overall HRQL. Its presence corresponds to a decrease of more than 5 points on the EQ-VAS. Another condition affecting several dimensions and overall HRQL was cardiac insufficiency. On the EQ-VAS its presence led to a decrease of more than four points, while it affected the dimensions self care, usual activities and mobility significantly. As expected, conditions like chronic low back pain and joint arthrosis were very influential factors on Pain/Discomfort. However, anemia had the strongest impact on pain/discomfort and was remarkably associated with mobility and usual activities.

Although the objective of our study was the analysis of the effect of multimorbidity on HRQL we want to point out the influence of social support on HRQL of patients suffering from multiple chronic conditions. Various studies have reported that low social support is a predictor for negative health outcomes such as increased mortality (e.g. [30]). We found similar results for HRQL with low social support being associated with worse overall HRQL and more problems in HRQL dimensions. This is in accordance with the results of Fortin et al. who showed that social support is associated with all dimensions of the SF-36 [8].

A potential limitation of our study is the composition of our study sample. Patients were recruited in a GP setting, and subjects residing in nursing homes were excluded, as well as patients suffering from severe diseases likely to be fatal within a short time. Consequently, our sample may represent patients with at most moderate disease severity. For this reason HRQL aspects specific for multimorbid patients suffering from severe conditions may not have been captured.

### Conclusion

The HRQL of multimorbid patients is affected by the number of conditions and the severity of these conditions. In our sample of elderly multimorbid patients, Parkinsońs disease had the strongest impact on overall HRQL, followed by rheumatism, depression and obesity. The specific dimensions of HRQL (mobility, self-care, usual activities, pain/discomfort, anxiety/depression) were affected by a great number of different conditions, especially depression, obesity, chronic low back pain, neuropathies and Parkinsońs disease.

Thus, the impact of chronic conditions on HRQL is complex and heterogeneous. Further studies should address the impact of disease combinations which require very large sample sizes as well as advanced statistical methods because of to the great number of possible combinations.
